# Advantages of L-3-[^18^F] fluoro-alpha-methyl tyrosine over 2-[^18^F]-fluoro-2-deoxyglucose in detecting liver metastasis during positron emission tomography scan

**DOI:** 10.1186/s40064-016-2212-7

**Published:** 2016-05-13

**Authors:** Sayaka Kodaira, Takahito Nakajima, Yukiko Arisaka, Azusa Tokue, Tetsuya Higuchi, Yoshito Tsushima

**Affiliations:** Department of Diagnostic Radiology and Nuclear Medicine, Gunma University Graduate School of Medicine, 3-39-22, Showa, Maebashi, Gunma 371-8511 Japan; Department of Molecular Imaging, Gunma University Graduate School of Medicine, 3-39-22, Showa, Maebashi, Gunma 371-8511 Japan

**Keywords:** Liver metastasis, L-3-[^18^F] fluoro-alpha-methyl tyrosine, 2-[^18^F]-fluoro-2-deoxyglucose, Positron emission tomography

## Abstract

**Purpose:**

We aimed to assess the usefulness of positron emission tomography (PET) using the amino acid tracer L-3-[18F] fluoro-alpha-methyl tyrosine (FAMT) in detecting metastatic liver lesions compared with 2-[18F]-fluoro-2-deoxyglucose (FDG).

**Methods:**

We included 24 patients with liver metastases who underwent both FDG-PET/computed tomography (CT) and FAMT-PET/CT. Maximum standardized uptake value (SUVmax) and tumor-to-liver parenchymal (T/L) ratio were analyzed to evaluate the correlation between FDG and FAMT uptakes in metastatic liver lesions; adenocarcinoma (AC, n = 21), squamous cell carcinoma (SCC, n = 23), neuroendocrine tumor (NET, n = 9), and carcinoid tumor (CAR, n = 6).

**Results:**

We detected 59 lesions on performing either FDG-PET or FAMT-PET. NETs had significantly lower T/L ratios for FAMT (median, 1.00; range, 0.86–1.34) compared with those for FDG (median 2.86; range 1.70–6.13, p < 0.01). CAR tumors tended to reveal lower T/L ratios for FDG (median 1.10; range 0.78–1.92) than those for FAMT (median 1.80; range 0.80–2.34). Comparison of T/L ratios of SCC and AC revealed that FAMT in the metastatic liver lesions of SCC was higher than those of AC (p < 0.05).

**Conclusion:**

FAMT-PET could detect metastatic liver lesions from various cancers, except NET.

## Background

Involvement of the liver as a site of cancer metastasis causes significant problems in morbidity and mortality. Because liver dysfunction due to the poor control of liver metastasis would shorten patient survival period, the assessment of metabolic function of the liver with metastasis is an important prognostic tool (Wiering et al. 2005; Bonanni et al. 2014; Fernandez et al. 2004).

Positron emission tomography (PET) using an ^18^F-labeled glucose analog, 2-[^18^F]-fluoro-2-deoxyglucose (FDG) is the most sensitive imaging method to detect malignant lesions that utilize glucose at greater rates than normal tissue because of an increase in both glucose transport and metabolism through the glucose transporter (GLUT1) (Juweid and Cheson 2006). Because metastatic liver lesions have little or no glucose-6-phosphatase, FDG is trapped within the cell (Mamede et al. 2005; Okazumi et al. 1992). Therefore, FDG-PET scans reveal that metastatic liver lesions have a high FDG uptake.

Many clinical studies revealed the feasibility of FDG-PET for diagnosing, staging, treatment monitoring, and detecting recurrent malignant tumors (Fischer et al. 2009; Pieterman et al. 2000; Kostakoglu and Goldsmith 2003). The detection of liver metastasis from colorectal and other gastrointestinal cancers has been evaluated in many reports; most of them addressed the very high sensitivity of FDG-PET in detecting liver metastasis compared with other modalities, such as computed tomography (CT) and magnetic resonance imaging (MRI), by the administration of conventional contrast agents (Kinkel et al. 2002; Bipat et al. 2005). Currently, MRI using gadolinium-ethoxybenzyl-diethylenetriamine pentaacetic acid (Gd-EOB-DTPA) has shown very high detectability for hepatic lesions. However, FDG-PET still has the advantage for detecting liver lesions. In contrast, the sensitivity of FDG-PET was not good, particularly for small liver lesions. The reasons would be the poor spatial resolution of PET and that FDG uptake in normal parenchymal tissue would obscure small liver lesions without high-FDG uptake. In addition, differentiation between inflammation and tumor lesions is difficult for FDG-PET (Nishii et al. 2013; Yamada et al. 1998; Morita et al. 2013). Nevertheless, FDG-PET still plays an important role in detecting metastatic liver lesions in a clinical setting.

It also is well known that amino acid tracers also accumulate in cancer lesions. In contrast to FDG, amino acid tracers do not accumulate in inflammatory lesions and the specificity of amino acid tracers to malignant lesions is reliable compared with that of FDG(Inoue et al. 1999, 2001). Tumor cells require amino acid transporters such as L-amino acid transporter type 1 (LAT1) (Kaira et al. 2008; Namikawa et al. 2015). In our facility, we developed L-3-[^18^F] fluoro-alpha-methyl tyrosine (FAMT) as an amino acid PET tracer(Tomiyoshi et al. 1997). The specific accumulation of FAMT in malignant tumors has been evaluated in a clinical setting and has been demonstrated to be useful in the diagnosis of various types of malignant tumors. With regard to the detection of lesions in the liver, because the most investigated amino acid tracers, ^11^C-methionine (MET) and anti-1-amino-3-18F-fluorocyclobutane-1-carboxylic acid (FACBC), accumulate in both cancerous and normal liver cells, their uptake in the normal liver tissue is strong enough to obscure the accumulation in metastatic lesions. In contrast, FAMT uptake in a normal liver has been known to be low, making FAMT a good candidate to detect metastatic liver lesions.

In this study, the accumulations of FDG and FAMT in metastatic liver lesions were compared and correlated with their respective histological features.

## Methods

### Patients

This retrospective study included 24 patients (17 men and seven women; aged 32–85 years; mean 67 years) with advanced cancer complicated by liver metastases who had undergone both FDG-PET/CT and FAMT-PET/CT between August 2007 and November 2014. The inclusion criteria were as follows: (1) <1-month interval between FDG-PET/CT and FAMT-PET/CT scans and (2) none of the metastatic lesions received treatment. Patients with duodenal pleomorphic sarcoma (n = 1), malignant lymphoma (n = 2), and lung small cell carcinoma (n = 3) were excluded from this study because the number of patients with liver metastasis for each group was only one. Blood samples were taken before tracer injection and an acceptable blood sugar level (<200 mg/dl) was confirmed. This study was reviewed and approved by our institutional review board, and informed consent was obtained from all patients.

### PET tracer administration

Both FDG and FAMT were synthesized in the cyclotron facility of our institute; FAMT was synthesized according to the method of Tomiyoshi et al. Briefly, L-alpha-methyltyrosine was fluorinated by [^18^F]-acetylhypofIuoride, and the separation and purification of FAMT were performed by a remote control system (Tomiyoshi et al. 1997). Patients were intravenously injected with FAMT (5 MBq/kg) and FDG (5 MBq/kg) after fasting for >6 h.

### Image acquisition

PET/CT images were acquired at 1 h (60 ± 5 min) after injection using a Discovery STE PET/CT scanner (GE Healthcare, Milwaukee, WI) or a Biograph 16 PET/CT scanner (Siemens, Malvern, PA) with 700-mm field of view (FOV) and a slice thickness of 3.27 mm. Three- dimensional (3D) data acquisition was performed for 3 min per bed position, followed by image reconstruction with the 3D-ordered-subsets expectation maximization (3D-OSEM) method. Segmented attenuation was corrected by X-ray CT (140 kV, 120–240 mAs) to produce 128 × 128 matrix images. CT images were reconstructed using a conventional filtered back projection method. Axial full width at half-maximum (FWHM) at 1 cm from the center of FOV was 5.6 mm; the z-axis FWHM at 1 cm from the center of FOV was 6.3 mm. Intrinsic system sensitivity was 8.5 cps/kBq for 3D acquisition. Both the PET scanners were regularly calibrated with a phantom and their standardized uptake value (SUV) accuracy was routinely evaluated to ensure that the values produced were comparable. Patients were scanned from the thigh to the head in the arms-down position. No intravenous contrast material was administered for CT scanning. Limited breath holding at normal expiration was used during CT to avoid motion-induced artifacts and allow co-registration of CT and PET images in the area of the diaphragm.

### Data analysis

PET/CT images acquired using FDG and FAMT were interpreted by a single experienced nuclear medicine physician and were analyzed using a syngo MI Workplace (VA60C, Siemens AG, Munich, Germany). The resolution of the reconstructed images was approximately 5 mm at FWHM.

To evaluate the distribution of both the PET tracers, the regions of interest (ROIs) were manually placed on each tumor lesion in the liver with assistance of CT images on the same slices, as well as on the normal liver parenchyma, back muscle, and mediastinum, which represented a blood pool by two experienced nuclear medicine physicians. SUVs in ROIs were calculated using the following formula: SUV = [radioactive concentration in ROI (MBq/g)]/[injected dose (MBq)/patient’s body weight (g)].

Side-by-side review and analysis of radioactive images were performed to confirm that SUV was derived from the same lesion on both the PET scans. In this study, we employed SUVmax, which was defined as the peak SUV on the pixel with the highest count within ROI.

All tumor lesions were categorized by the histological examination of the primary lesion or the liver biopsy specimen as adenocarcinoma (AC); squamous cell carcinoma (SCC); neuroendocrine tumor (NET; i.e., pheochromocytoma, malignant endocrine tumor, and paraganglioma); and carcinoid tumor (CAR). To evaluate the correlation between FDG and FAMT uptakes in liver lesions, three SUVmax-related parameters were analyzed; these were (1) SUVmax itself, (2) tumor-to-liver parenchymal (T/L) ratio, and (3) tumor-to-blood pool (T/B) ratio.

### Statistical analysis

The correlation between FDG and FAMT uptakes in the metastatic liver lesions in each histological group was evaluated by linear regression analysis. The Mann–Whitney U test was performed to evaluate the differences between each SUVmax parameter of FAMT and FDG. For all statistical analyses, p value <0.05 was considered statistically significant.

## Results

The primary tumors were esophageal carcinoma (n = 10), lung carcinoma (n = 7), pancreatic carcinoma (n = 2), rectal carcinoid (n = 2), and one patient each for primary lesions in the gingiva, adrenal gland, and carotid body (Table 1). The median interval between FDG-PET and FAMT-PET was 7.6 days (range 1–30 days).

**Table 1:** Characteristics of patients with liver metastasis

No.	Age (years)	Sex	Primary tumor	Lesion number	Size (cm)	Histological type
1	59	M	Pancreas	3	1.8–7.2	Adenocarcinoma
2	64	F	Lung	3	2.4–3.1	Adenocarcinoma
3	83	M	Rectum	3	2.0–11.0	Carcinoid
4	67	F	Rectum	3	8.8–12.4	Carcinoid
5	61	M	Esophagus	2	2.1–3.1	Squamous cell carcinoma
6	73	M	Esophagus	3	1.8–7.7	Squamous cell carcinoma
7	60	F	Lung	1	5.3	Adenocarcinoma
8	70	M	Esophagus	3	1.0–1.7	Squamous cell carcinoma
9	73	M	Lung	3	1.9–2.0	Adenocarcinoma
10	66	M	Lung	3	2.0–3.0	Squamous cell carcinoma
11	53	M	Lung	1	3.1	Adenocarcinoma
12	77	M	Esophagus	3	1.6–2.2	Endocrine cell carcinoma
13	57	M	Esophagus	3	7.5–11.8	Adenocarcinoma
14	65	F	Adrenal gland	3	1.1–2.6	Malignant pheochromocytoma
15	67	M	Esophagus	1	2.3	Squamous cell carcinoma
16	67	F	Esophagus	3	8.3–10.6	Squamous cell carcinoma
17	74	M	Lung	3	1.6–2.0	Adenocarcinoma
18	85	M	Gingiva	3	2.4–4.8	Squamous cell carcinoma
19	32	M	Lung	1	1.6	Adenocarcinoma
20	53	F	Carotid body	3	2.4–4.4	Paraganglioma
21	84	M	Esophagus	1	2.8	Squamous cell carcinoma
22	81	F	Esophagus	3	2.6–6.3	Squamous cell carcinoma
23	56	M	Pancreas	3	4.3–5.2	Adenocarcinoma
24	78	M	Esophagus	1	10.0	Squamous cell carcinoma

### Tracer uptake in metastatic liver lesions and liver parenchymal tissue

A total of 59 lesions were detected on either FDG-PET or FAMT-PET in this study. Two lesions were not detected in FDG-PET/CT but were detected in FAMT-PET/CT. Fourteen lesions demonstrated opposing results with those of PET/CT; for these lesions, ROIs were carefully drawn while referring to PET with another tracer and enhanced CT images. On the basis of histological examinations, primary lesions were divided into four forms (Table 1): SCC (n = 23), AC (n = 21), NET (n = 9), and CAR (n = 6).

**Fig. 1:**
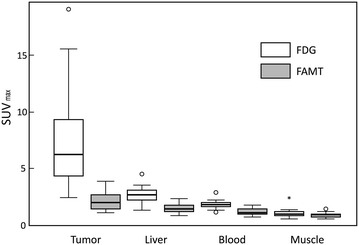
Comparison of FDG and FAMT by SUVmax in each area. FDG uptake showed significant differences between metastatic liver lesions (tumor) and the other areas without tumor involvement, specifically, normal liver parenchyma (liver), blood pool (blood), and muscle (p < 0.001, respectively). FDG uptake was significantly higher than that of FAMT in all areas. In the comparison with tumor and liver, SUVmax of FDG in tumor (median 6.16; range 2.40–19.01) was significant higher than that of FAMT (median 1.85; range 1.02–3.85). *SUV* standard uptake value, *FDG* 2-[^18^F]-fluoro-2-deoxyglucose, *FAMT* L-3-[^18^F] fluoro-alpha-methyl tyrosine

SUVmax in these lesions ranged from 2.40 to 19.01 (median 6.16) for FDG and from 1.02 to 3.85 (median 1.85) for FAMT. As shown in Fig. 1, uptake of FAMT in metastatic lesions was significantly lower than that of FDG (p < 0.001), whereas liver parenchymal tissue demonstrated an almost double SUVmax of FDG compared with that of FAMT (p < 0.001); the median SUVmax of ^18^F-FDG and ^18^F-FAMT in liver parenchymal tissue was 2.61 (range 1.36–4.50) and 1.39 (0.88–2.32), respectively.

### Correlation between FDG and FAMT by each parameter

**Fig. 2:**
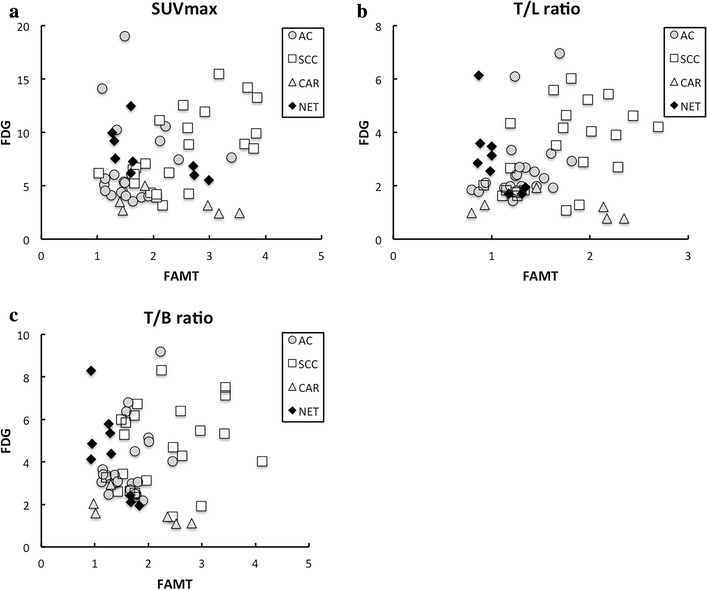
Dot plots of SUVmax, T/L ratio, T/B ratio, and T/M ratio. *X* axis shows the SUVmax of FAMT and the *Y* axis shows the SUVmax of FDG. No significant correlations of total lesions in **a** SUVmax, **b** T/L ratio, and **c** T/B ratio were observed (r = 0.167, p = 0.207; r = 0.208, p = 0.114; r = 0.043, p = 0.744, respectively). T/L ratio differed according to histological feature. *SUV* standard uptake value, *FDG* 2-[^18^F]-fluoro-2-deoxyglucose, *FAMT* L-3-[^18^F] fluoro-alpha-methyl tyrosine, *T/L* tumor-to-liver parenchymal, *T/B* tumor-to-blood pool, *SCC* squamous cell carcinoma, *AC* adenocarcinoma, *NET* neuroendocrine tumor and pheochromocytoma, *CAR* carcinoid tumor

Scatter plots between FDG and FAMT by each parameter are shown in Fig. 2. No significant correlations in SUVmax (r = 0.167, p = 0.207); T/L ratio (r = 0.208, p = 0.114); and T/B ratio (r = 0.043, p = 0.744) were observed. The distributions indicated that (1) CAR lesions had low FDG uptake and relatively high FAMT uptake; (2) NET showed low FAMT uptake and relatively high FDG uptake; (3) the T/L ratios of SCC tended to be higher than those of AC. Only two lesions of AC showed high FDG uptakes compared with SCC; these two lesions were derived from esophageal cancer.

### Distribution of T/L ratio in each histological category

**Fig. 3:**
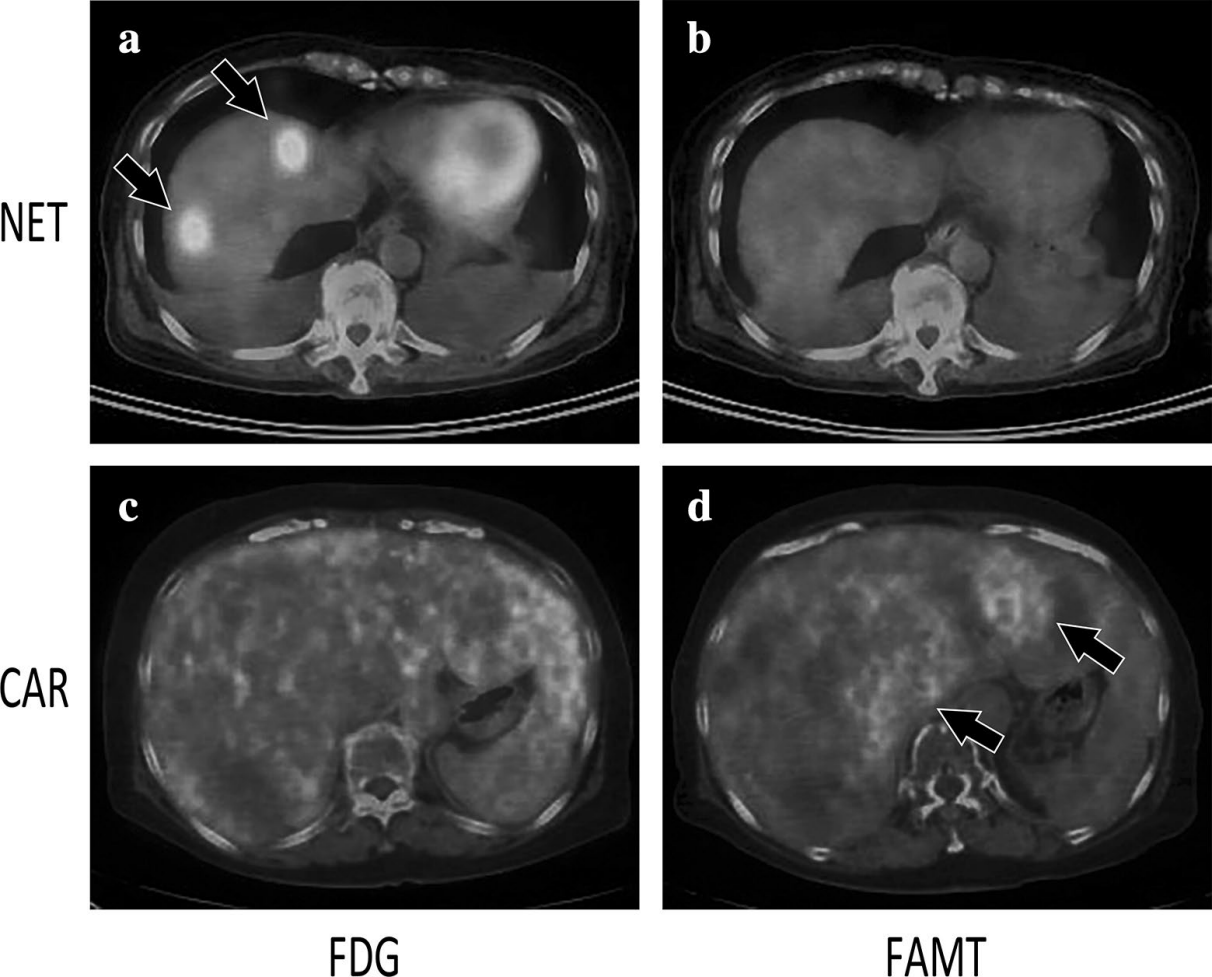
PET/CT fusion images with either FDG or FAMT administration for the same patients with NET or CAR. For a patient with NET, FDG PET images showed high spotty accumulations (*arrow*) in the liver (**a**). No FAMT uptakes were seen in the liver (**b**). For a patient with CAR, FDG PET showed diffuse uptake for normal liver parenchyma without uptakes for tumor lesion in the liver (**c**). FAMT PET showed some faint massive uptakes (*arrow*) of FAMT in the liver (**d**). *FDG* 2-[^18^F]-fluoro-2-deoxyglucose, *FAMT* L-3-[^18^F] fluoro-alpha-methyl tyrosine, *T/L* tumor-to-liver parenchymal, *CAR* carcinoid tumor, *NET* neuroendocrine tumor and pheochromocytoma

**Fig. 4:**
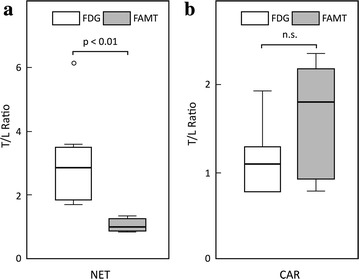
Comparison between FDG an FAMT in terms of T/L ratio in NET lesions and CAR lesions. **a** T/L ratio of FAMT uptake was significantly lower than that of FDG in the group of NET lesions (p < 0.01). **b** T/L ratio of FAMT (median 1.80) was slightly higher than that of FDG (median 1.10), but not significant (p = 0.345). *FDG* 2-[^18^F]-fluoro-2-deoxyglucose, *FAMT* L-3-[^18^F] fluoro-alpha-methyl tyrosine, *T/L* tumor-to-liver parenchymal, *CAR* carcinoid tumor, *NET* neuroendocrine tumor and pheochromocytoma

The distribution of T/L ratio demonstrated some tendencies depending on the histological category, particularly for NET and CAR (Fig. 3). NETs had significantly lower T/L ratios for FAMT uptake (median 1.00; range 0.86–1.34) compared with those for FDG uptake (median 2.86; range 1.70–6.13) (p < 0.01, Fig. 4a). CAR tumors tended to reveal lower T/L ratios for FDG uptake (median 1.10; range 0.78–1.92) than those for FAMT uptake (median 1.80; range 0.80–2.34), but this was not significant (p = 0.345, Fig. 4b).

### Comparison of FDG and FAMT uptakes between SCC and AC (Figs. 5, 6)

The T/L ratio of SCC had median values of 3.50 (range 1.09–6.02) on FDG-PET and 1.76 (range 0.92–2.69) on FAMT-PET. The T/L ratio of AC had median values of 2.28 (range 1.43–6.96) on FDG-PET and 1.26 (range 0.80–1.81) on FAMT-PET. Comparison of T/L ratios of SCC and AC revealed that FAMT uptake in the metastatic liver lesions of SCC was higher than that of AC (p < 0.01). In contrast, FDG uptakes of SCC and AC were not significantly different (p = 0.235).

**Fig. 5:**
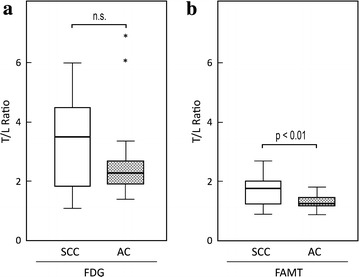
Comparison of T/L ratios in FDG and FAMT between SCC and AC. **a** FDG uptakes in liver metastasis from SCC were higher than those from AC (p < 0.05). **b** FAMT uptakes for SCC and AC were not significantly different (p = 0.235). *FDG* 2-[^18^F]-fluoro-2-deoxyglucose, *FAMT* L-3-[^18^F] fluoro-alpha-methyl tyrosine, *T/L* tumor-to-liver parenchyma, *T/B* tumor-to-blood pool, *SCC* squamous cell carcinoma, *AC* adenocarcinoma

**Fig. 6:**
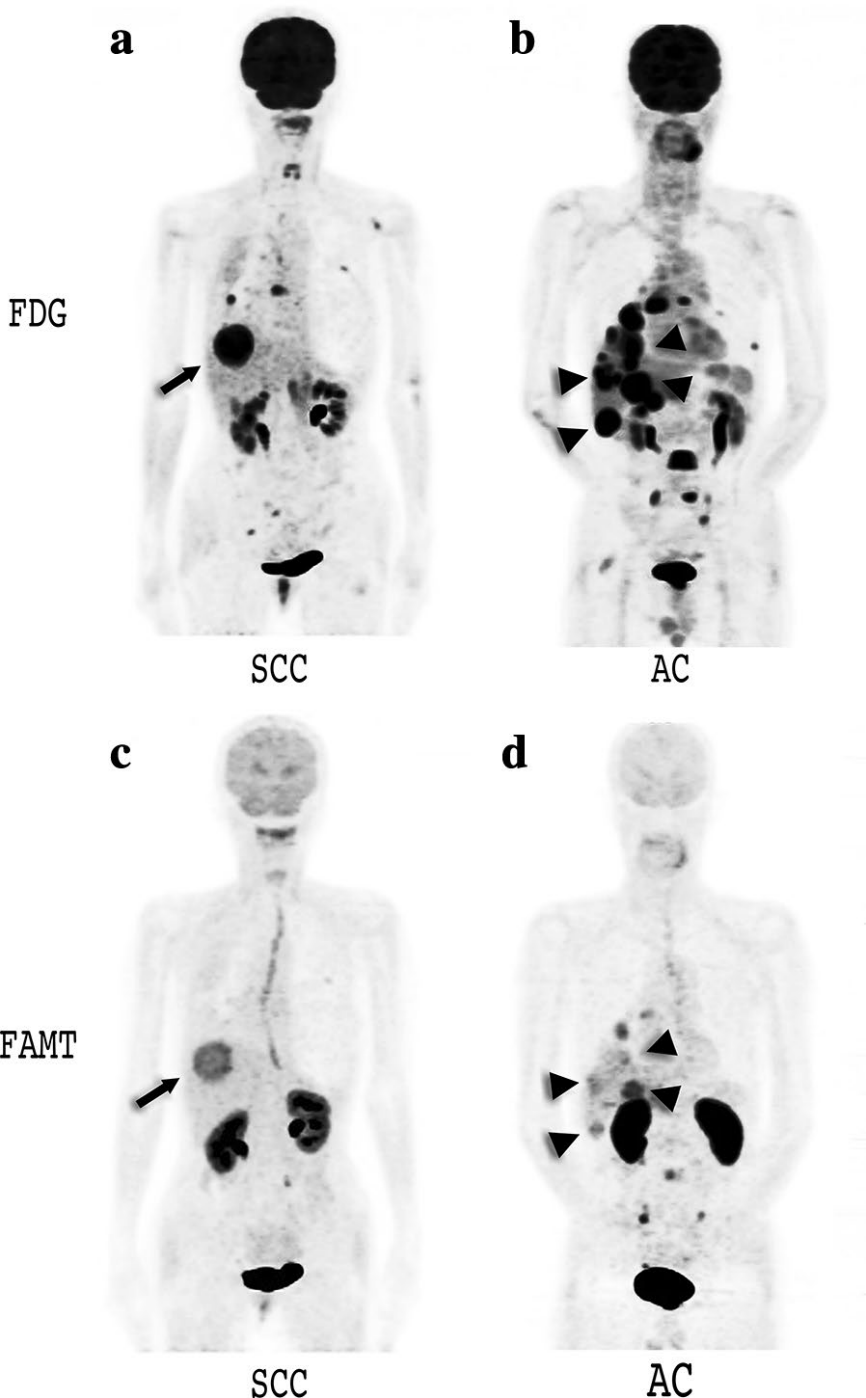
PET images of patients with either SCC or AC. PET images were acquired from two patients with metastases of SCC (**a**, **c**) and AC (**b**, **d**). High FDG uptakes were seen in the liver (**a**, **b**) while weak to high FAMT uptakes were seen in the liver (**c**, **d**). Normal liver uptakes of FAMT were weaker than those of FDG. *FDG* 2-[^18^F]-fluoro-2-deoxyglucose, *FAMT* L-3-[^18^F] fluoro-alpha-methyl tyrosine, *SCC* squamous cell carcinoma, *AC* adenocarcinoma

## Discussion

The respective uptake of FAMT was lower in both metastatic liver lesions and normal tissues compared with that of FDG. However, most liver metastases were recognized on FAMT-PET images, except NET lesions. Differential diagnosis among various tissues using T/L ratio might be useful, particularly for comparison between SCC and AC on FAMT-PET. In FDG and FAMT combinations, tissues tended to be distributed on the basis of their histology (Fig. 2).

Both FDG and FAMT uptakes for SCC were relatively higher than those for AC. Only two lesions of AC in one patient showed higher SUVmax compared with that in SCC; however, excluding these two lesions, the T/L ratio of FDG revealed no significant difference between SCC and AC (p = 0.063). This patient was the only case of esophageal adenocarcinoma. We speculated that this cancer lesion might have included adenosquamous components. Further investigations of LAT1 expression based on histology will be required to demonstrate the utility of FDG-PET or FAMT-PET for differentiating between SCC and AC. FDG uptakes of CAR tumors had relatively low T/L ratios, resulting in difficulty to differentiate metastatic liver lesions from liver parenchymal tissues on FDG-PET. In contrast, CAR tumors were detected on FAMT-PET and FAMT-PET was superior to FDG-PET in this point.

In this study, FDG uptake in the liver parenchymal tissues ranged from 1.36 to 4.50, and the median value of SUVmax (2.60) was already over the upper limit of the general threshold between benign and malignant lesions. FDG uptake in the liver parenchyma was higher than that of FAMT because glucose was metabolized and stored in the liver. In general, hepatocytes have glucose-6-phosphatase that can reverse FDG-6-phosphate to FDG, thus reducing FDG uptake in the hepatocyte (Torizuka et al. 1995; Khan et al. 2000; Yen et al. 2004). Therefore, FDG accumulation was relatively lower than what was expected of a glucose analog.

The differences between FDG and FAMT may be because of not only metabolism but also retention. Because FDG was taken up by GLUT1 and trapped into cells after phosphorylation, it accumulated in a linear fashion and was eventually retained. Conversely, after FAMT transport into cells by LAT1, relatively rapid clearance of FAMT can be observed in even tumor cells; this means that LAT1 function of FAMT transport was not one way but was reversible. These mechanisms may explain the lower SUVmax of FAMT.

Alpha-methylation of FAMT reduces liver accumulation and increases renal excretion, whereas the analog of FAMT, l-tyrosine, has been well known to be one of the aromatic amino acids that are metabolized in the liver. This is an advantage of FAMT over the most commonly studied MET, which strongly accumulates in both the normal and metastatic liver tissues (Wiriyasermkul et al. 2012). Another analog of tyrosine and a well-known amino acid tracer, O-(2-^18^F-fluoroethyl)-l-tyrosine (FET), has been shown to have low accumulation in murine liver and a higher specificity than FDG (Ishiwata et al. 2004). Although FET would be a good candidate for detecting liver metastasis, most reports on its application were for brain tumors, and there has been no report on liver metastasis.

Wiriyasermkul et al. (2012) revealed that FAMT had two potential benefits: one was the reduced uptake in the liver and another was the specific transport via LAT1 without the need to go through other types of amino acid transporters, such as the system ASC amino acid transporter-2 (ASCT2). The details of the mechanisms for uptake of FAMT or methionine in normal liver are not clear; however, FAMT is transported only via L-amino acid transporter-1 (LAT1), while methionine is transported via LAT-1, LAT-2, and some other transporters (Singhal et al. 2008). LAT1 specificity of FAMT may increase the possibility of detecting metastatic liver lesions.

Nevertheless, because FDG strongly accumulated in the metastatic liver lesions, T/L ratios were still higher than those of FAMT. In this study, T/L ratio indicated the specific features based on histological origins. Although FAMT uptake was detected in metastatic liver lesions from NETs, FDG accumulation was very high (median; 7.28, ranging from 5.50 to 12.45). Our clinical experiences revealed that FAMT-PET had a difficulty in the detecting pheochromocytoma lesions (unpublished data), whereas FDG had an advantage. In contrast, the detection of liver metastasis from CAR tumors using FDG-PET was difficult because FDG uptake was low; thus, for these lesions, FAMT-PET could be more useful. According to the International Union Against Cancer classification of neuroendocrine tumors, CAR belongs to G1 and NET belongs to G2 or G3. Because the uptake of FDG generally reflects the grading of tumor differentiation, FDG-PET should have shown higher accumulations in NET than in CAR. Low-grade tumors, such as NET, show a high uptake of FDG compared with well-differentiated tumors such as CAR. In our case, two lesions were detected using only FAMT-PET not FDG-PET.

The limitation of this study was that patients with colorectal cancer were not included. Although most metastatic liver lesions from colorectal cancer would be AC, our AC group included metastasis from lung cancers. However, some reports revealed high expressions of GLUT1 and high FDG uptake in SCC (Yen et al. 2004; Fletcher et al. 2008). Our comparison between SCC and AC may not be different from that for colorectal cancer. FAMT-PET was performed not for conventional clinical examinations but for study purposes; this study did not include metastatic liver lesions from colorectal cancers. Therefore, the number of patients who underwent both FDG-PET and FAMT-PET was small, and this would be a limitation for statistical analysis. In further investigations, the time of PET acquisition after tracer administration can be changed to an earlier time. It would have an advantage with obtaining high FAMT uptakes in liver lesions. However, the high T/L ratio would be required to depict liver lesions more clearly. Because of the background signals, including normal liver uptakes is another important factor and should be investigated for an optimal time.

## Conclusion

There is no general advantage in use of FAMT-PET over that offered by FDG for detecting liver metastasis. However, since FAMT-PET could detect metastatic liver lesions from many types of cancers, except NET, histological tendencies were shown by comparisons between PET studies of FDG and FAMT.
